# The Blackheath and Charlton Cottage Hospital

**Published:** 1889-01-19

**Authors:** 


### THE BLACKHEATH AND CHARLTON COTTAGE HOSPITAL.

FROM A CORRESPONDENT.

THE building which it is proposed to erect, and a plan of which is being circulated with an appeal for funds, has been designed by Mr. J. Mechelen Rogers, whose design was chosen out of a number of others submitted in a limited competition. The building consists of a centre block of two storeys in height, with wings of one storey. The centre block contains committee-room, dispensary, kitchen offices, and store on the ground floor, with bed-rooms for matron, nurses, and servants on the floor above. The corridor, which bisects this block, separates the kitchen, scullery, and store from the committee-room, entrance, and dispensary. The corridor is continued at each side of the centre block until it reaches the ward blocks. One half the corridor is provided with windows on both sides, on the other, one side is entirely taken up with a waiting-room and the operating-room. The ward blocks each contain a general ward for eight beds, a single-bed ward, and a nurses' room, and in a wing placed near the entrance to the ward a w.c., sink, bath-room, and lobby. The lobby is evidently intended to serve the purpose of ward scullery, and is marked "stove" on the plan. The bath-room is so small that it would be quite impossible to bathe a patient requiring to be lifted in and out of the bath. There is no direct access from the ward to the w.c., etc., so that patients going to the latter must all come out of the ward and through a passage; and this corridor is so planned that a skylight has to be provided just outside the ward-door in order to get light. At the back of the administration block there is an enclosed yard, and on one side of this, less than ten feet distant from the kitchen widows, is a long narrow shed with apparently no other means of ventilation than the door, which faces the kitchen widows. The amount to which competitors were limited was, we believe, under £2,000. It would be interesting to learn if the building represented in this plan is really to be erected and finished for this sum. The architect is, we are told, or at any rate was, one of the draughtsmen employed in the office of the Director-General of Fortifications at the War Office. Was there any special reason for this selection?

